# An intra-operative checklist for low-impact robotic urologic surgery: a three-round J-ERUS/YAU Delphi consensus

**DOI:** 10.1007/s11701-026-03983-5

**Published:** 2026-09-30

**Authors:** Gabriele Sorce, Maria Peraire-Lores, Luigi Nocera, Mike Wenzel, Marco Paciotti, Angelo Mottaran, Gianluca Spena, Sij Hemal, Fabrizio Di Maida, Ruben De Groote, Carlo Andrea Bravi, Filippo Turri, Federico Lavagno, Edward Lambert, Carlo Gandi, Paolo Dell’Oglio, Christoph Würnschimmel, Stefano Tappero, Marcio Covas Moschovas, Federico Piramide, Danny Darlington Carbin Joseph, Nikolaos Liakos, Ahmed Eraky, Marco Oderda, Xavier Ruiz-Plazas, Helena Ascaso-Til, Alejandro Juarez-Sanchez, José Segarra-Tomàs, Violeta Petrelli-Tain, Josep Maria Santillana-Altamira, Manuel Prados-Saavedra, Nicolae Crisan, Alessandro Larcher, Giorgio Ivan Russo, Alberto Breda, Alex Mottrie, Iulia Andras

**Affiliations:** 1 Urology Service, Institut de Recerca Biomèdica Catalunya Sud, Hospital Universitari Joan XXIII de Tarragona, Universitat Rovira i Virgili, C/Dr. Mallafré Guasch, 4, ES-43007 Tarragona, Spain; 2 Spedali Civili of Brescia, Urology, Brescia, Italy; 3 University Hospital Frankfurt, Urology, Frankfurt, Germany; 4 IRCCS Humanitas Research Hospital, Urology, Milano, Italy; 5 IRCCS Azienda Ospedaliero-Universitaria di Bologna Policlinico Sant’Orsola, Urology, Bologna, Italy; 6 IRCCS Fondazione G. Pascale, Urology, Napoli, Italy; 7 Norris Comprehensive Cancer Center, Urology, Los Angeles, USA; 8 Careggi Hospital, Urology, Firenze, Italy; 9 Department of Urology, AZORG Hospital, Aalst, Belgium; 10 Northampton General Hospital, Urology, Northampton, UK; 11 A. Gemelli University Hospital Foundation IRCCS, Urology, Roma, Italy; 12 A.O.U. Citta della Salute e della Scienza, Urology, Torino, Italy; 13 ASST Grande Ospedale Metropolitano Niguarda, Urology, Milano, Italy; 14 Luzerner Kantonsspital, Urology, Lucerne, Switzerland; 15 AdventHealth Global Robotics Institute, Urology, Celebration USA; 16 San Luigi Gonzaga Hospital, Urology, Orbassano, Italy; 17 Royal Surrey NHS Foundation Trust, Urology, Guildford, UK; 18 Department of Urology, Medical Faculty of the University of Freiburg, Medical Centre of the University of Freiburg, Freiburg, Germany; 19 Department of Urology, Icahn School of Medicine at Mount Sinai, New York, NY USA; 20 Anaesthesiology Service, Institut de Recerca Biomèdica Catalunya Sud, Hospital Universitari Joan XXIII de Tarragona, Tarragona, Spain; 21 Iuliu Hatieganu University of Medicine and Pharmacy, Urology, Cluj-Napoca, Romania; 22 Department of Urology, IRCCS San Raffaele Scientific Institute, Milan, Italy; 23 Urology Section, Department of Surgery, University of Catania, Catania, Italy; 24 Department of Urology, School of Medicine, Biruni University, Istanbul, Turkey; 25 Department of Urology, Fundació Puigvert, Autonomous University of Barcelona, Barcelona, Spain

**Keywords:** Robotic surgical procedures, Low-impact surgery, Delphi technique, Postoperative pain, ERAS, Intraoperative care

## Abstract

Enhanced recovery pathways emphasise peri-operative pharmacology, whereas many modifiable intra-operative practices remain under-standardised and supported by heterogeneous evidence. Standardising these practices is central to low-impact robotic surgery, aiming to minimise avoidable procedural and physiological stress, and may improve early recovery, including post-operative pain. A three-round, online modified Delphi was conducted within the J-ERUS/YAU network, inviting 21 experts (19 urologists, 2 anaesthetists). Round-1 presented 21 items (5-point Likert). A priori thresholds—blinded to panellists—were: Include = median ≥ 4 with interquartile range (IQR) ≤ 1; Exclude = median < 3; otherwise = revote. Rounds-2/-3 re-rated non-consensus items and validated new items proposed from R1 free-text; at each revote, the prior round’s median and IQR were shown. Late submissions were excluded in Rounds 1 and 2. R1 analysed 20 valid responses: 5 Include, 2 Exclude, 14 to revote. R2 analysed 19 responses and re-rated 14 carried plus 9 new items from R1 suggestions: 16 reached Include, 7 to revote. R3 analysed 16 responses: 2 reached Include, 5 without consensus. In total, 23 items met Include (5 R1 + 16 R2 + 2 R3). The checklist integrates pharmacologic/anaesthetic measures (multimodal analgesia; regional techniques as appropriate; structured anti-PONV prophylaxis) with non-pharmacologic practices across surgical (pressure-point padding; low pneumoperitoneum/valveless insufflation) and environmental (active temperature management) domains. This Delphi produced a 23-item consensus checklist that standardises modifiable intra-operative practices underpinning low-impact robotic urologic surgery. Prospective implementation should evaluate effects on recovery outcomes, including post-operative pain, opioid use and PONV.

**Supplementary Information:**

The online version contains supplementary material available at https://doi.org/10.1007/s11701-026-03983-5.

## Introduction

Post-operative pain after robotic urologic procedures remains clinically relevant despite minimally invasive access and the diffusion of enhanced recovery after surgery (ERAS) principles. This persistence suggests that the impact of an operation reflects not only the platform used but how the procedure is conducted—a perspective increasingly described as low-impact robotic surgery [1] — the recognition that the conduct of the operation itself, from positioning and insufflation to operative duration and desufflation, is a modifiable determinant of recovery rather than merely the setting in which pharmacological management takes place. We treat this as an emerging concept rather than an established framework, and this consensus seeks to make it operational by targeting the intra-operative steps through which that impact is largely determined. ERAS emphasises peri-operative pharmacology and pathway organisation; by contrast, several modifiable intra-operative practices—spanning anaesthetic management, surgical conduct and the operating-room environment—remain under-standardised. The evidence supporting them is scarce and at times conflicting, and in consequence their contribution is often underestimated at the console and left to individual habit rather than codified. Growing data in robotic urology suggest that low-pressure pneumoperitoneum may reduce early post-operative pain, although effects on opioid consumption are less consistent [2–4]. At the same time, positioning-related neuropathies and pressure injuries have been reported after robotic pelvic surgery, underscoring the importance of positioning discipline and padding [5–7]. Many of these measures are simple, low-cost and time-neutral. Dedicated multidisciplinary consensus documents for robotic surgery have also been called for, particularly to support anaesthesia-surgery coordination and peri-operative planning [8]. The Delphi method offers a systematic means to synthesise expert judgement where evidence is dispersed and practice varies, using transparent thresholds for consensus and structured iteration. We therefore convened a Junior European Robotic Urology Society/Young Academic Urologists (J-ERUS/YAU) modified Delphi to identify and standardise intra-operative practices underpinning low-impact robotic urologic surgery and to translate them into a practical, evidence-informed checklist.

## Materials and methods

### Study design and scope

We conducted a three-round, online modified Delphi with the objective of producing an intra-operative checklist for robotic urologic surgery, following previous methodology [9, 10]. The J-ERUS/YAU coordinated the process. The target construct was deliberately comprehensive, spanning pharmacologic/anaesthetic practices and non-pharmacologic measures within the surgical and environmental domains.

### Panel, eligibility and governance

We invited 21 experts (19 urologists, 2 anaesthetists) from seven European countries and the USA. Eligibility required documented experience in robotic urology and/or anaesthesia for robotic surgery, involvement in ≥ 100 procedures per year, and affiliation or collaboration with J-ERUS/YAU. A steering group (urologists and anaesthetists) oversaw item wording, survey logistics and adherence to the protocol. Participation was voluntary and anonymous. Panel composition is summarised in Table 1.

**Table 1:** Composition and governance of the multidisciplinary Delphi panel

Characteristic	Value
Panel size (invited / R1 / R2 / R3)	21 / 20 / 19 / 16
Urologists	19
Anaesthetists	2
Countries represented	7 European countries + USA
Eligibility – annual robotic caseload	≥ 100 procedures/year
Affiliation	EAU–YAU/ERUS and network collaborators
Steering group	Urologists and anaesthetists (item wording, logistics, protocol adherence)

### Item development and Round-1 instrument

The initial pool of 21 items was derived from focused literature appraisal and steering-group input. Items were drafted to capture intra-operative factors plausibly related to post-operative pain and were reviewed for clarity, redundancy and actionability. The appraisal was performed by three authors (GS, MPL, LN) as a non-systematic review of the literature on intra-operative determinants of recovery after robotic surgery, without a predefined search protocol or formal screening; the pool is therefore expert-curated and evidence-informed rather than the product of a systematic or scoping review. In Round-1 (R1) panellists rated each item’s clinical relevance on a 5-point Likert scale (1 = not relevant; 5 = highly relevant) and could propose additions or refinements via free text.

### Consensus framework and iteration

Before data collection we pre-specified—and kept blinded to panellists—the thresholds for classification: Include if the item’s median rating was at least four and the interquartile range (IQR) no greater than one; Exclude if the median was below three; otherwise the item was carried forward to revote. These criteria are in line with commonly adopted Delphi criteria that use the IQR as a measure of dispersion [11, 12]. Items meeting Include or Exclude exited the process. Between rounds, structured controlled feedback was provided: at each revote the prior round’s group median and IQR were displayed for every item to support calibrated judgement while preserving anonymity. In addition, free-text comments collected at each round were reviewed by the steering group and used both to refine item wording and to introduce new candidate items proposed by panellists; these entered the following round and were rated by the full panel on the same scale and against the same a priori thresholds as the original pool. Nine such items emerged from Round-1 free-text and entered Round-2, and several existing items were re-worded before re-rating. Round-2 (R2) therefore re-rated non-consensus items from R1 together with these newly introduced and revised items. Round-3 (R3) re-rated only items that remained without consensus after R2.

### Quality control, handling of late submissions and statistics

Surveys were time-stamped; each round was closed when the following round was launched, and later submissions were not retro-applied (one in Round 1, two in Round 2). All 16 responses received in Round 3, the final round, were analysed. Data were anonymised. Missingness was handled per item; the number of ratings (N) is reported for each item and round. For each item/round we computed the median and IQR (Q3–Q1) and applied the consensus rules above. We summarised classification counts by round and retained item-wise medians, IQRs and Ns for tabulation.

## Results

### Participation and dataset

**Fig. 1:**
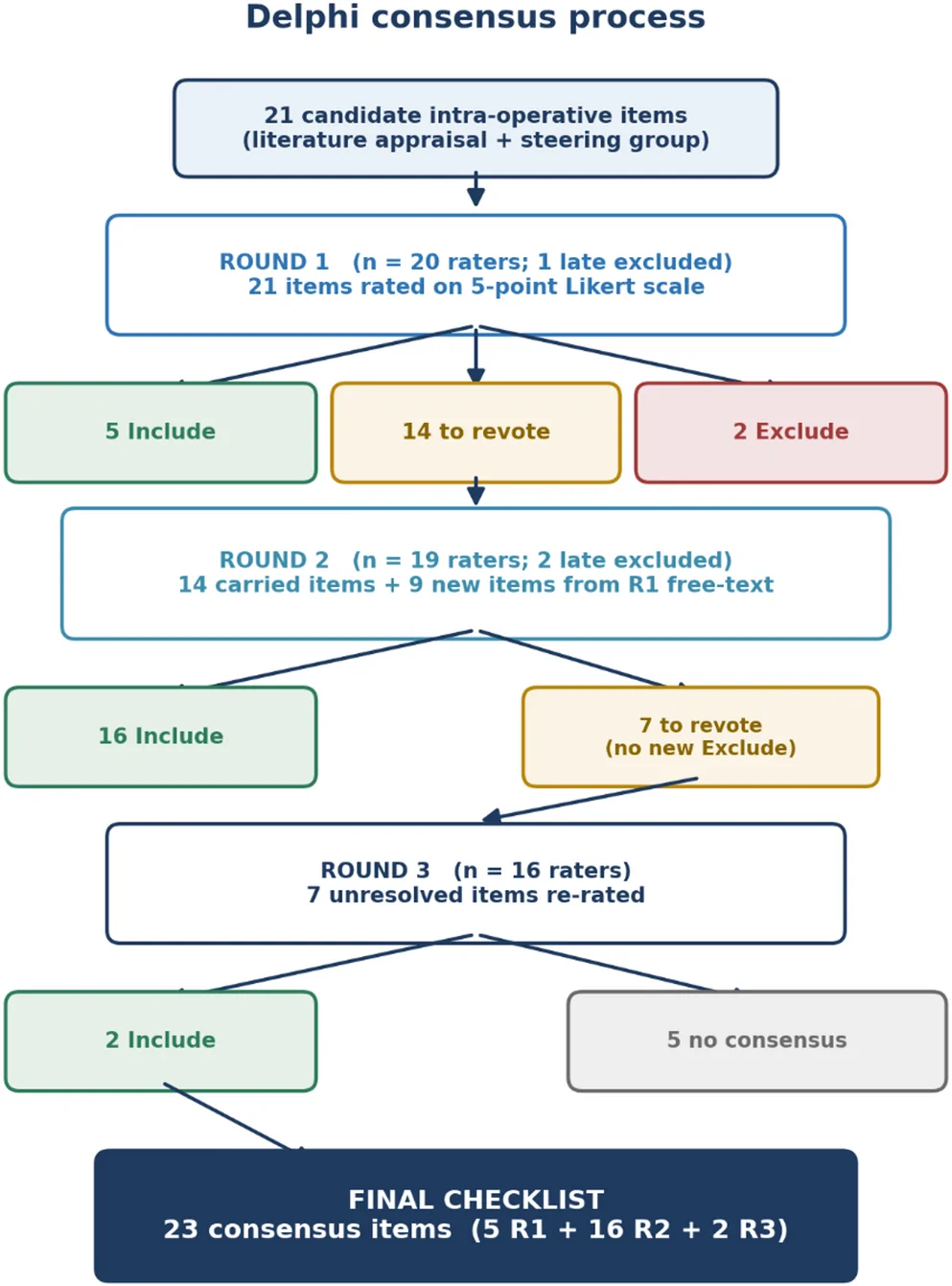
Flow of items through the three Delphi rounds. Counts reflect a priori consensus thresholds (Include: median ≥ 4 and IQR ≤ 1; Exclude: median < 3; otherwise revote). Late submissions were excluded in Rounds 1 and 2 (R1 n = 20, R2 n = 19, R3 n = 16)

R1, R2 and R3 analysed 20, 19 and 16 valid responses respectively, after excluding late submissionsin Rounds 1 and 2. Attrition was not differential by discipline, as both anaesthetists completed all three rounds. Sensitivity analyses including the late responses excluded from Rounds 1 and 2 showed that only the round at which consensus was reached was affected, with no Include item reclassified. The flow of items across rounds is summarised in Fig. 1, and item-level results in Table 2.

**Table 2:** Item-level results across rounds (median / interquartile range / number of raters)

Domain	Item	R1 med/IQR/*N*	R2 med/IQR/*N*	R3 med/IQR/*N*	Outcome
Anaesthetic-pharm.	Early intra-operative analgesic administration †	—	4.0 / 1.00 / 19	—	Include
	Multimodal intra-operative analgesia †	—	4.0 / 1.00 / 19	—	Include
	Regional anaesthesia (TAP/PVB/QL) in multimodal strategy †	—	4.0 / 1.00 / 19	—	Include
	Standardised anti-PONV prophylaxis †	—	4.0 / 1.00 / 19	—	Include
	TAP block	4.0 / 1.00 / 20	—	—	Include
	Trocar-site local anaesthetic infiltration	3.5 / 1.00 / 20	3.0 / 1.00 / 19	3.0 / 1.25 / 16	No cons.
Positioning	Anti-slip carepad (avoid shoulder braces, Trendelenburg) †	—	4.0 / 2.00 / 19	4.0 / 0.25 / 16	Include
	Arms slightly flexed and pronated	3.5 / 1.00 / 20	4.0 / 1.00 / 19	—	Include
	Avoid limbs over hard surfaces or cables	4.0 / 1.00 / 20	—	—	Include
	Check pressure on IV/monitoring lines	4.0 / 1.00 / 20	—	—	Include
	Check pressure zones (shoulder, flank, limbs)	5.0 / 1.00 / 20	—	—	Include
	Foam roll for axillary support	3.5 / 1.25 / 20	4.0 / 1.00 / 19	—	Include
	Legs in stirrups then lowered to horizontal	4.0 / 2.00 / 20	4.0 / 1.00 / 19	—	Include
	Neck flexed rather than extended	3.5 / 2.00 / 20	4.0 / 1.00 / 19	—	Include
	Procedure-specific positioning recommendations †	—	4.0 / 1.00 / 19	—	Include
	Systematic gel/jelly padding on pressure points †	—	4.0 / 1.00 / 19	—	Include
	Table flexed without kidney rest	4.0 / 1.00 / 20	—	—	Include
	Avoid raised kidney rest unless clinically required	3.0 / 2.00 / 20	3.0 / 1.00 / 19	3.0 / 0.00 / 16	No cons.
	Contralateral arm on overarm board	3.5 / 2.00 / 20	4.0 / 1.50 / 19	3.5 / 1.00 / 16	No cons.
Pneumoperitoneum	Insufflation pressure < 10 mmHg	4.0 / 2.00 / 20	5.0 / 1.00 / 19	—	Include
	Pneumoperitoneum time minimised	4.0 / 2.00 / 20	5.0 / 1.00 / 19	—	Include
	Valveless trocar system (e.g., AirSeal) †	—	4.0 / 1.50 / 19	4.0 / 0.00 / 16	Include
	Active desufflation (manual pressure + suction)	3.0 / 1.25 / 20	3.0 / 0.50 / 19	3.0 / 0.25 / 16	No cons.
	Low initial insufflation rate (1 L/min) †	—	3.0 / 1.00 / 19	3.0 / 0.25 / 16	No cons.
Operative course	Avoid routine abdominal drains (increase discomfort)	4.0 / 1.25 / 20	4.0 / 0.50 / 19	—	Include
	Prolonged operative duration ‡	—	4.0 / 1.00 / 19	—	Include
	Operative time < 180 min	4.0 / 2.00 / 20	—	—	Reform.
	Drain to evacuate residual CO₂ (reduce shoulder pain)	2.0 / 1.25 / 20	—	—	Exclude
Environmental	Stable intra-operative temperature	4.0 / 2.00 / 20	4.0 / 1.00 / 19	—	Include
	Surgeon–anaesthesiologist communication	4.0 / 2.00 / 20	4.0 / 1.00 / 19	—	Include
	Reduced intra-operative noise	2.0 / 1.25 / 20	—	—	Exclude

Empty cells indicate items not rated in that round (already classified, not yet introduced, or reformulated). Shading: green = Include, red = Exclude, grey = no consensus after three rounds, blue = reformulated. † item proposed by panellists through Round-1 free-text and introduced in Round-2; ‡ item reformulated after Round-1 feedback. The candidate item “operative time < 180 minutes” was not carried unchanged: after Round-1 free-text feedback it was reformulated into “prolonged operative duration,” which then reached Include in Round-2; both rows are shown, adjacently, to preserve the audit trail

### Round-1

Of the 21 items presented, 5 met the Include criterion (median ≥ 4 with IQR ≤ 1), 2 met Exclude (median < 3), and 14 were carried forward to revote. The 2 R1 Exclude items—reduced intra-operative noise and the use of an abdominal drain to evacuate residual CO₂/fluids to mitigate shoulder pain—did not achieve sufficient group-level relevance.

### Round-2

Following structured feedback (display of R1 median/IQR for each revoted item) and validation of R1 suggestions, 16 additional items reached Include and 7 remained without consensus. No new Exclude was observed in R2. Nine of the items rated in R2 were newly introduced from Round-1 free-text suggestions—anti-slip carepad use, systematic gel padding, procedure-specific positioning recommendations, valveless trocar systems, low initial insufflation rate, multimodal analgesia, regional anaesthesia within a multimodal strategy, early analgesic administration, and standardised anti-PONV prophylaxis—while the original “operative time < 180 min” item was reformulated as “prolonged operative duration.”

### Round-3 and final classification

Of the 7 items re-submitted for a third-round decision, 2 additional items achieved Include and 5 remained without consensus. Across rounds, 23 items satisfied the Include criterion (5 from R1, 16 from R2, 2 from R3). Content-wise, the final checklist integrates pharmacologic/anaesthetic measures—multimodal intra-operative analgesia, structured anti-PONV prophylaxis and regional techniques such as transversus abdominis plane (TAP) blocks where appropriate—with non-pharmacologic practices across surgical (pressure-point padding; strategies consistent with low pneumoperitoneum pressure, including valveless insufflation where available) and environmental (active temperature management) domains. Overall, the items define a practical intra-operative standard intended to minimise avoidable procedural impact and to support consistent early recovery pathways, including pain-related outcomes. The 23 consensus items, presented as 22 operational actions grouped by peri-operative phase, are shown in Table 3.

**Table 3:** Final intra-operative checklist for low-impact robotic urologic surgery, structured for real-time operating-room use. The 23 consensus items are presented as 22 operational actions grouped into three peri-operative phases, each with a primary responsible role

Phase	Consensus item	Primary responsible role	Round
Phase 1 — Pre-induction and positioning	Check pressure zones (shoulder, flank, limbs)	Circulating nurse / bedside assistant	R1
	Avoid limbs over hard surfaces or cables	Circulating nurse / bedside assistant	R1
	Check pressure on IV/monitoring lines	Anaesthetist / circulating nurse	R1
	Table flexed without kidney rest	Surgeon / circulating nurse	R1
	Arms slightly flexed and pronated	Circulating nurse / bedside assistant	R2
	Foam roll for axillary support	Circulating nurse / bedside assistant	R2
	Legs in stirrups then lowered to horizontal	Circulating nurse / bedside assistant	R2
	Neck flexed rather than extended	Anaesthetist	R2
	Systematic gel/jelly padding on pressure points	Circulating nurse / bedside assistant	R2
	Anti-slip carepad (avoid shoulder braces in Trendelenburg)	Circulating nurse / bedside assistant	R3
	Procedure-specific positioning recommendations applied	Surgeon	R2
Phase 2 — Insufflation and pre-docking	Insufflation pressure < 10 mmHg	Surgeon / bedside assistant	R2
	Valveless trocar system (e.g., AirSeal) where available	Surgeon / bedside assistant	R3
	Regional anaesthesia (TAP / PVB / QL block) within a multimodal strategy	Anaesthetist	R1 + R2 †
	Multimodal intra-operative analgesia	Anaesthetist	R2
	Early intra-operative analgesic administration	Anaesthetist	R2
	Standardised anti-PONV prophylaxis	Anaesthetist	R2
	Active temperature management initiated	Anaesthetist / circulating nurse	R2
	Surgeon–anaesthesiologist communication on plan and expected duration	Surgeon and anaesthetist	R2
Phase 3 — Operative course, desufflation and emergence	Pneumoperitoneum time minimised	Surgeon	R2
	Prolonged operative duration reconsidered (prompt at ≈ 150 min steep Trendelenburg and ≈ 210 min operative time)	Surgeon and anaesthetist	R2
	Avoid routine abdominal drains	Surgeon	R2

“Round” indicates the Delphi round in which each item reached the Include threshold (R1 = consensus on first rating; R2/R3 = consensus after structured feedback). † The R1 item “TAP block” and the R2 item “regional anaesthesia (TAP/PVB/QL) within a multimodal strategy” both reached consensus and are merged here into a single operational recommendation to avoid redundancy at the point of use; the separate item-level results are retained in Table 2. Adherence may be defined per phase as the proportion of applicable items explicitly verified

## Discussion

Post-operative pain remains a meaningful outcome after robotic urologic surgery, and the variation in practice across centres suggests that intra-operative contributors are not yet addressed consistently. Many decisions taken during the operation — spanning anaesthetic management, surgical conduct and the theatre environment — still rely on local habit, reflecting both a heterogeneous evidence base and the limited extent to which intra-operative practice is codified in a form that can be taught, audited and compared. Against this background, our Delphi is primarily a standardisation effort. It defines what a multidisciplinary panel considered worth doing routinely to support low-impact robotic surgery, minimising avoidable procedural and physiological impact.

The main contribution of the checklist is standardisation rather than novelty. The final items translate common-sense and selectively supported measures into explicit, consistently applicable expectations, and several already rest on emerging, if uneven, evidence. Importantly, the items differ in their evidence base: some are supported by randomised trials or systematic reviews, others by prospective or observational data or by formal consensus documents, and several rest primarily on expert judgement (Table 4). The checklist should therefore be read as a graded rather than uniform set of recommendations, and this heterogeneity signals where evidence is most needed. Low-pressure and ultra-low pneumoperitoneum have been associated with lower early post-operative pain after robotic prostatectomy and in systematic review data, although effects on opioid outcomes remain less consistent [2–4, 13]. Valveless insufflation appears to act along the same axis, with randomised and meta-analytic data reporting lower early and shoulder pain than conventional systems, again without a clear opioid-sparing effect [14, 15]. The emphasis on positioning and padding is likewise not arbitrary. In a prospective study of awake volunteers, slightly flexed and pronated arms, a flexed neck, a foam axillary roll and a flexed table without a raised kidney rest all produced significantly lower pain scores than their alternatives, observations that map directly onto several of our positioning items [16]; this is complemented by a consistent body of work on positioning-related neuropathies, in which prolonged operative and Trendelenburg time, higher body mass index (BMI) and comorbidity recur as risk factors [6, 17]. Avoiding routine drainage reflects a shift in robotic practice, with evidence showing less discomfort without more complications [18]. The environmental and team-related items are less often formalised but are not unsupported, since poor intra-operative communication and suboptimal theatre organisation have been linked to longer operative times and worse surgical performance [19, 20]. Anaesthetic items such as multimodal analgesia and structured anti-PONV prophylaxis are consistent with the broader direction of peri-operative optimisation captured in multidisciplinary consensus documents for robotic and abdominal surgery [8, 21].

**Table 4:** Evidence base supporting each consensus item, classified as RCT / SR (randomised trials or systematic reviews/meta-analyses), Observational (prospective or observational data, including systematic reviews of observational studies), Consensus (formal multidisciplinary consensus or guideline documents), or Expert consensus (panel judgement). A single representative reference is given for each item; a dash denotes items resting on panel consensus

Consensus item	Evidence base	Representative reference
TAP block	RCT / SR	*Dal Moro 2018* ^23^
Early intra-operative analgesic administration	Consensus	*Corcione 2018* ^8^
Multimodal intra-operative analgesia	Consensus	*Corcione 2018* ^8^
Regional anaesthesia (TAP/PVB/QL) in multimodal strategy	RCT / SR	*Cacciamani 2019* ^22^
Standardised anti-PONV prophylaxis	Consensus	*Aceto 2019* ^21^
Avoid limbs over hard surfaces or cables	Observational	*Cornelius 2021* ^6^
Check pressure on IV/monitoring lines	Expert consensus	*—*
Check pressure zones (shoulder, flank, limbs)	Observational	*Cornelius 2021* ^6^
Table flexed without kidney rest	Observational	*Ginsburg 2018* ^16^
Arms slightly flexed and pronated	Observational	*Ginsburg 2018* ^16^
Foam roll for axillary support	Observational	*Ginsburg 2018* ^16^
Legs in stirrups then lowered to horizontal	Observational	*Ginsburg 2018* ^16^
Neck flexed rather than extended	Observational	*Ginsburg 2018* ^16^
Procedure-specific positioning recommendations	Expert consensus	*—*
Systematic gel/jelly padding on pressure points	Expert consensus	*—*
Anti-slip carepad (avoid shoulder braces, Trendelenburg)	Expert consensus	*—*
Insufflation pressure < 10 mmHg	RCT / SR	*Abaza 2022* ^3^
Pneumoperitoneum time minimised	Expert consensus	*—*
Valveless trocar system (e.g., AirSeal)	RCT / SR	*Feng 2021* ^14^
Avoid routine abdominal drains	RCT / SR	*Territo 2023* ^18^
Prolonged operative duration	Observational	*Göksu 2025* ^17^
Stable intra-operative temperature	Consensus	*Aceto 2019* ^21^
Surgeon–anaesthesiologist communication	Observational	*Pasquer 2024* ^19^

A more instructive case is regional analgesia, where the evidence is genuinely mixed. Randomised trials in robot-assisted prostatectomy have reported lower pain scores and reduced opioid demand with a TAP block [22, 23], and a further series supports it after robotic surgery more broadly [24]; yet a randomised comparison found no advantage of a TAP block over simple wound infiltration [25], and a recent Cochrane review found no clinically meaningful effect on pain or opioid consumption [26]. The panel nevertheless retained regional analgesia as an option within a multimodal strategy. This captures a recurring feature of the work — consensus can standardise reasonable practice even where randomised evidence is heterogeneous, while signalling precisely where that evidence is still needed. The practical implication is that low-impact robotic surgery is not only a technological attribute but a reproducible intra-operative process. The checklist shifts the conversation from which platform is used to how the operation is conducted, and makes those choices measurable. Although pressure-area protection and padding discipline are not specific to robotic surgery, steep Trendelenburg and the inaccessibility of the patient after docking make lapses in them both more likely and far harder to correct. Two anaesthetic items, TAP block and regional anaesthesia within a multimodal strategy, reached consensus separately because the panel first rated a specific technique and then endorsed the broader principle; they are merged into a single recommendation, so that the 23 items reaching Delphi consensus are presented as 22 operational actions in Table 3, with the item-level results retained in Table 2.

The iterative structure also let item content mature rather than be merely accepted or rejected. A candidate item specifying an operative time below 180 min was relevant on average but widely dispersed in Round-1 (median 4, IQR 2), and a panellist questioned the evidence for that cut-off; it was therefore reformulated as a qualitative statement on prolonged operative duration, which then reached consensus. The reformulated item aligns better with the available evidence than the original threshold, since prolonged operative and Trendelenburg time recur as risk factors for positioning-related injury across several series, and where thresholds have been proposed they relate to nerve injury rather than pain and differ from the discarded value — for instance, operative and Trendelenburg-time cut-offs of approximately 212 and 157 min for positional nerve injury [17]. A generic statement on prolonged duration is therefore better supported than a single pain-specific threshold. For implementers who require an auditable trigger, we suggest treating the published nerve-injury thresholds as pragmatic prompts rather than limits: a verbal check at approximately 150 min of steep Trendelenburg and at approximately 210 min of total operative time, at which the team explicitly reconsiders tilt angle, padding and pressure points. These values derive from positional nerve-injury data rather than from pain outcomes, and are offered as a starting point for local adaptation and prospective evaluation rather than as validated cut-offs.

Two domains are addressed only indirectly. Prolonged steep Trendelenburg with pneumoperitoneum reduces functional residual capacity and compliance, promotes atelectasis and raises airway pressures, effects that accumulate with time and interact directly with the consensus items on pneumoperitoneum pressure and duration; the panel nevertheless generated no item on ventilatory management or limitation of tilt angle, which reflects its composition rather than the importance of the domain. Operative technique itself is the second: meticulous dissection, minimisation of traction and thermal injury, and haemostatic discipline are plausibly among the strongest determinants of the physiological impact of an operation, yet no such item was proposed, most likely because technical quality resists expression as a binary, auditable entry. The checklist therefore addresses the modifiable conditions under which surgery is performed, not the quality of the dissection, which remains the domain of training and performance assessment.

Equally important, the checklist creates a shared language for teams. By turning intra-operative behaviours into defined checkpoints, it becomes easier to teach good practice, document adherence and identify variation. This is relevant for training and quality improvement, since items map onto ERUS/YAU educational activities such as rehearsing positioning and padding, standardising pneumoperitoneum set-up, or embedding anti-PONV checks within anaesthesia routines. Documenting adherence also supports benchmarking across centres: the checklist is not meant to mandate a single method, but to ensure that modifiable steps are explicitly considered and addressed.

The pattern of residual disagreement is informative. The items that failed to reach consensus after three rounds were not scattered across domains but clustered around the management of residual CO₂ and the finer parameters of insufflation — active desufflation, low initial insufflation rate and trocar-site infiltration — where supporting evidence is weakest. For these items medians stayed intermediate while IQRs narrowed across rounds, indicating convergence on uncertainty rather than persistent disagreement. Read this way, the unresolved items map the questions that prospective work should address first.

Implementation should be pragmatic and minimally disruptive. Rather than a stand-alone protocol, the checklist can be embedded into existing safety processes as a short, role-assigned pauses at a moment of maximal actionability. Documentation can remain light, with tick-box completion and a rationale when an item is not feasible, enabling audit-and-feedback cycles without substantial administrative burden. Adoption will also depend on local context: institutional resources, anaesthetic protocols and access to technologies such as valveless insufflation vary between centres, so some items may require adaptation rather than uniform application. Future studies should therefore evaluate not only clinical outcomes but also adherence, since implementation fidelity will be central to determining its true impact.

A single pause covering all items would risk checklist fatigue. The instrument is therefore structured as three short, role-assigned checkpoints — before induction and positioning, before insufflation and docking, and at desufflation and emergence — which can be nested within the safety pauses already in use in the operating room, such as the WHO Surgical Safety Checklist, rather than duplicating them. Table 3 presents the checklist in this form, and adherence can be defined per phase as the proportion of applicable items verified.

Several results warrant careful interpretation. Two items were excluded early and several required repeated revoting before inclusion, reflecting genuine uncertainty and the interdependence of surgical, anaesthetic and environmental factors. Delphi consensus is not causal proof, so the checklist should be viewed as a standardisation tool and a platform for prospective evaluation rather than as an endpoint in itself. The key next step is multicentre implementation with prospective measurement of early recovery outcomes — pain trajectories, rescue opioid use, PONV, readiness for mobilisation and length of stay — together with fidelity and balancing measures relevant to intra-operative physiology and workflow. Process evaluation should address barriers and document local adaptations while preserving comparability. The checklist also offers opportunities beyond pain alone. By linking intra-operative actions to early outcomes, it may support analgesic stewardship and help reduce unwarranted variation in opioid exposure, although any such effect remains to be demonstrated prospectively. The same intra-operative factors are also relevant to the shift towards same-day discharge after robot-assisted procedures, where recovery must be compressed into hours and any avoidable source of pain, nausea or delayed mobilisation becomes limiting [28]. Because many items are low-cost and time-neutral, the economic implications may be favourable: even modest reductions in opioid use, PONV or length of stay could translate into value, while the main cost of adoption is implementation effort that can be folded into existing safety and quality programmes. The instrument is procedure-agnostic within robotic urology, allowing stratified evaluation by procedure type with a consistent process metric.

This work has limitations. First, the panel was small and predominantly surgical: two anaesthetists rated a checklist in which five of the twenty-three items are explicitly anaesthetic, and those items should therefore be regarded as provisional pending an anaesthesia-weighted extension of this work; profession-based weighting was not applied, which preserves anonymity but limits discipline-specific interpretation. Second, attrition left 16 respondents by Round 3, and because the items that never reached consensus cluster in insufflation and residual-CO₂ management, consensus bias cannot be formally excluded. Third, the item pool was not fixed a priori, since nine items entered in Round 2 from panel free text, although these were rated under the same rules as the original pool. Fourth, the consensus thresholds are conventional rather than externally validated [12, 27] and alternative cut-points might shift classifications near the margin. Finally, the checklist addresses only intra-operative, team-modifiable conditions and does not incorporate patient-reported priorities; Delphi consensus standardises process, but its clinical effect on impact-related outcomes, including pain, requires prospective evaluation.

## Conclusions

In summary, this three-round J-ERUS/YAU Delphi distils multidisciplinary expertise into a 23-item intra-operative checklist that complements ERAS by standardising modifiable pharmacologic and non-pharmacologic practices underpinning low-impact robotic urologic surgery. The instrument is designed for real-time operating-room use, provides a common language for training, audit and benchmarking, and offers a concrete scaffold for multicentre implementation. As an expert consensus, it standardises process rather than demonstrating clinical benefit: prospective studies should now assess fidelity and quantify effects on early recovery outcomes, including post-operative pain, opioid use and PONV, while monitoring relevant balancing measures.

## Supplementary Information

Below is the link to the electronic supplementary material.

Supplementary Material 1

## Data Availability

The datasets generated and analysed during the current study are available from the corresponding author upon reasonable request.
