# Supplementary material for: An intra-operative checklist for low-impact robotic urologic surgery: a three-round J-ERUS/YAU Delphi consensus
Source: J Robot Surg. 2026 Sep 30;20(1):1041. doi: 10.1007/s11701-026-03983-5 (PMC13627210; doi:10.1007/s11701-026-03983-5)
Supplement: Supplementary file 1 [file 11701_2026_3983_MOESM1_ESM.docx]

**Supplementary Figure 1**

Supplementary Figure 1. Heat-map of item median Likert ratings across rounds (cell labels: median and IQR). Rows are grouped by final classification and the colour scale runs from red (low relevance) to green (high relevance). Included items consolidate at median 4–5, whereas non-consensus items remain stable at median 3 with narrowing IQR, indicating genuine intermediate relevance rather than dispersion.
